# Potential greenhouse gas risk led by renewable energy crowding out nuclear power

**DOI:** 10.1016/j.isci.2022.103741

**Published:** 2022-01-07

**Authors:** Xiaoli Zhao, Zewei Zhong, Xi Lu, Yang Yu

**Affiliations:** 1 School of Economics and Management, China University of Petroleum, Beijing 102299, China; 2 Beijing Laboratory of Environmental Frontier Technologies, School of Environment, Tsinghua University, Beijing 100084, China; 3 State Key Joint Laboratory of Environment Simulation and Pollution Control and State Environmental Protection Key Laboratory of Sources and Control of Air Pollution Complex, Tsinghua University, Beijing 100084, China; 4 Institute for Interdisciplinary Information Sciences, Tsinghua University, Beijing 100084, China

**Keywords:** Energy resources, Energy policy, Energy sustainability, Energy systems, Energy flexibility

## Abstract

Increasing variable renewable energy (VRE) is one of the main approaches for greenhouse gas (GHG) mitigation. However, we find a GHG increase risk associated with increasing VRE: VRE crowds out nuclear power (VRECON) but cannot fully obtain the left market share, which is obtained by fossil energy. We developed an integrated dispatch-and-investment model to estimate the VRECON GHG-boosting effect in the Pennsylvania-New Jersey-Maryland Interconnection and the Electric Reliability Council of Texas. In the above two markets, VRECON could increase the annual GHG emission by up to 136 MTCO_2_eq totally. Furthermore, we find that the VRECON GHG-boosting effect can be mitigated by combining wind and solar power. We argue that, for GHG abatement, policymakers should require the proper mix of wind and solar power in renewable portfolio standards and control nuclear power’s retirement pace to match the progress of VRE growth.

## Highlights

• Variable renewable energy could boost greenhouse gas by crowding out nuclear power
• The size of greenhouse gas boost is impacted by wind and solar mix
• Renewable portfolio standards need to identify the mix of wind and solar power
• Nuclear power retirement pace and variable renewable energy growth shall be matched

## Graphical abstract

**Figure:**
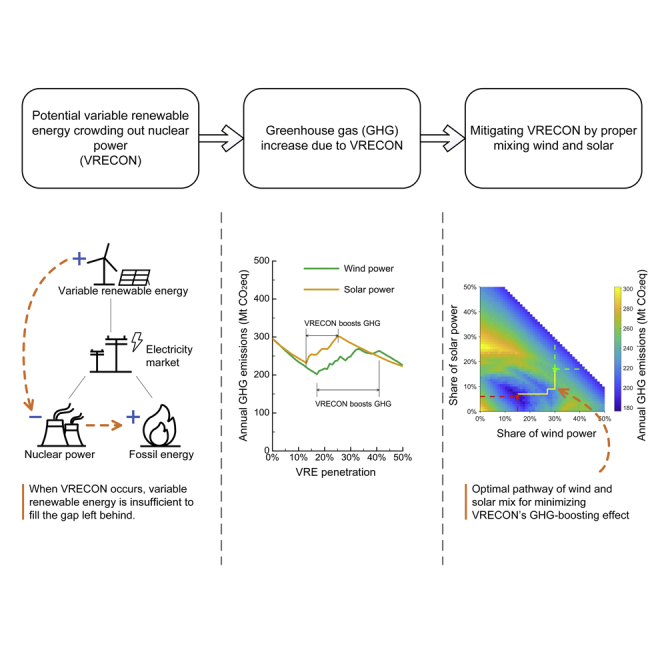

## Introduction

Renewable energy has been promoted to reshuffle electricity generation, thus working toward decarbonization (Hertwich et al., 2015; Pacala, 2004). Many countries have set ambitious targets for renewable energy penetration in the coming decades. For instance, the European Union purports to supply at least 32% of its electricity demand by renewable energy by 2030 (the European Parliament and the Council of the European Union, 2018). Maryland and New Jersey, in the United States, have targeted that their renewable portfolio standards (RPSs) for 2030 are up to 50% (National Conference of State Legislatures, 2020). In 2019, global renewable energy electricity generation reached 2108 TWh, equivalent to 1.27 times that of 2015 and to 23.2% of the world’s total electricity consumption in that year (IEA, 2021). Variable renewable energy (VRE), mainly wind power and solar power, are the main growth segments, accounting for 42.7% and 31.6%, respectively, of the growth in renewable energy generation (IEA, 2021).

However, in the process of pursuing VRE development goals, VRE would replace not only fossil energy but also low-carbon technologies, including nuclear power, since VRE will lead to increased volatility in net loads and steeper net load distribution curves (Figure S1). Some studies have theoretically pointed out that VRE can polarize the peak and off-peak net loads in some markets and decrease the number of peak-load hours, which threats the market shares of base-load generators (Nicolosi and Fürsch, 2009; Ueckerdt et al., 2013). A few very recent studies have noticed the correlation between VRE’s penetration and the decline of production and profit of nuclear power plants (Sovacool et al., 2020; Mills et al., 2020; Jenkins, 2018). Sovacool et al. (2020) found a negative correlation between renewable electricity production and nuclear electricity production based on a study across 125 countries over 25 years. Mills et al. (2020) summarized the reported impacts on operating revenues and profits for nuclear plants and found a decrease in revenue and operating profits for nuclear power plants as VRE penetration increases. Jenkins (2018) employed time series linear regression to empirically estimate the effect of various factors on the market price earned by 19 nuclear-generating stations located in the Pennsylvania-New Jersey-Maryland Interconnection (PJM). He found that declining natural gas prices, increasing wind power generation, and stagnant or declining electricity demand appear to be cutting the profitability of nuclear power producers in PJM.

Hence, the increasing VRE has raised concern about the early retirement of existing nuclear power capacity (Clemmer et al., 2018; EIA, 2018; U.S. Department of Energy, 2017). In a case study of nuclear power retirement, the increase of VRE was considered as one of the many reasons to retire the Diablo Canyon nuclear plant in California (Wiser et al., 2017), which implies that a potential effect of VRE crowding out nuclear power (VRECON) exists. Some theoretical analysis has noticed the risk of VRECON with the “screening curves” approach (Nicolosi and Fürsch, 2009; Ueckerdt et al., 2013). They noticed that VRE growth could lead to nuclear power capacity decrease. However, to the best of our knowledge, the risk of VRECON raising greenhouse gas (GHG) emissions has not attracted sufficient attention while the complex relation between VRECON and electricity system GHG emissions are ignored during the relative studies. It is unclear which penetration level of VRE will trigger the VRECON progress, and there is a lack of a method to verify which types of generators will replace nuclear power endogenously crowded out by VRE. The absence of the answers to the two questions prevents us from analyzing and assessing the VRECON’s impact on future GHG trajectory.

To identify the issue of VRECON rising GHG emissions, an integrated dispatch model and investment (retirement) decision model are needed. An hourly dispatch model is necessary to capture whether nuclear power or fossil energy loses market share when VRE penetration shrinks the demand for base-load generators. Once VRECON occurs, the hourly dispatch analysis also manifests whether VRE or fossil energy will occupy the market share left by crowded-out nuclear generators. Meanwhile, the hourly dispatch model should be paired with the model of annual investment and retirement of electricity generators, which reflects how VRECON reshapes the energy mix over various types of generators, and finally how VRECON influences GHG emissions.

We developed an integrated midterm electricity model of capturing the complex mechanism that VRECON impacts GHG emissions. Our model allows for endogenous investment and retirement of electricity generators. Thus, by analyzing the dynamics and interaction between electricity hourly dispatch and the decision on generators' investment and retirement, we can calibrate how much nuclear power will be crowded out with different VER penetration levels. Our model can also answer the question about what types of power sources will take the market share left by crowding out nuclear generators in the market competition. In the STAR Methods part, we provide the details of our model.

We apply our model to examine the following three sequential questions: (1) At what level of VRE penetration will VRECON start? (2) When VRECON occurs, is VRE sufficient to fill the gap left behind? If not, what will happen to the fossil fuel power generation and GHG emissions of the electricity system? (3) How does the VRE mix (the proportion of wind and solar power generation to total power generation) fluctuate the progress of VRECON as well as the GHG emissions? These three questions enable us to analyze the process of VRECON and its impact on GHG emissions. PJM and the Electric Reliability Council of Texas (ERCOT) are taken as examples since they have been used as cases studies related to electricity systems and both have nuclear power (Fell and Linn, 2013; Linn and Shih, 2019; Lueken and Apt, 2014; Weis et al., 2015).

The answers to the previous three questions are keys for the decarbonization progress of the electricity system that have a large share of nuclear power. We found that, when VRECON occurs, the share of nuclear power decreases faster than the increase of VRE; that is to say, the share of fossil energy increases and so do GHG emissions. For instance, we found VRECON will cause GHG emission increases in both PJM and ERCOT. Furthermore, VRECON can be mitigated by adjusting the proportion of wind power and solar power, which implies that it is a better choice for policymakers not only to stipulate the VRE share in the total power generation in their RPS but also require the mix of VRE for mitigating VRECON, and it is important to promote the coordinated progress of VRE development and nuclear power retirement in order to realize electricity system decarbonization.

## Results

### The potential risk of VRECON boosting GHG

**Figure 1:**
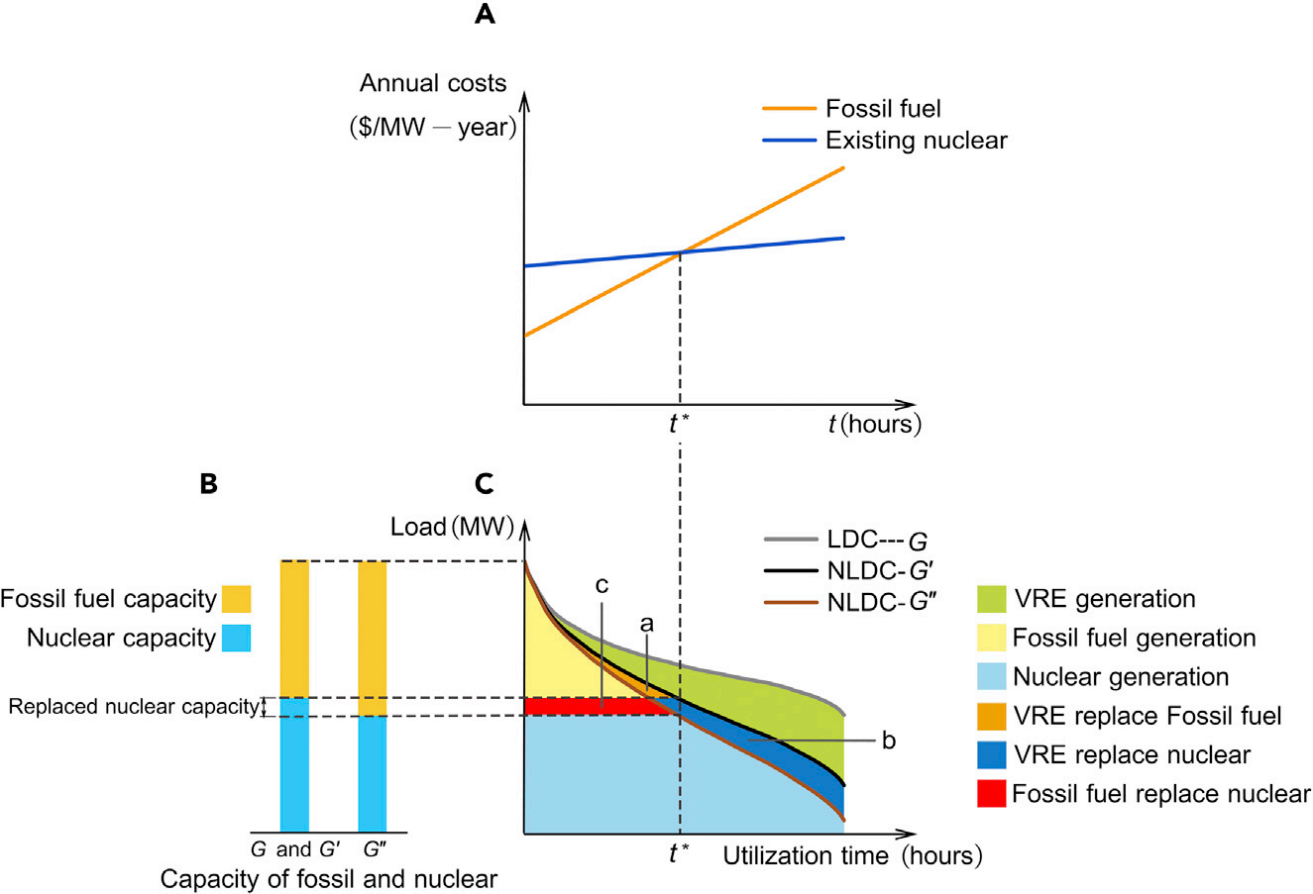
The impact of VRE expansion on nuclear power retirement and GHG emissions

To demonstrate the concept of VRECON and its impact on GHG emissions, we constructed an analytical framework as shown in Figure 1, which is based on the studies of Ueckerdt et al. (2013) and Nicolosi and Fürsch (2009). This analytical framework is also known as the "screening curves" approach. It was developed for the regulated electricity systems, but it is still useful for understanding perfect competitive markets with perfect foresight (Biggar and Hesamzadeh, 2014; Stoft, 2002). This approach aims at minimizing the total cost of electricity supply, displaying corresponding investment and scheduling plans (Batlle and Rodilla, 2013; Biggar and Hesamzadeh, 2014).

Figure 1A shows the relationship between the electricity generation cost and utilization time of a 1-MW generator of fossil energy and nuclear power in a year. The intercept of the curve represents the fixed cost, and the slope represents the marginal cost. Compared with fossil fuel power generation, nuclear power generation has a relatively high fixed cost and low marginal cost. In Figure 1C, the (net) load duration curves (LDC/NLDC) show all individual (net) load hours in a subsequent order. The abscissa of the points on LDC/NLDC shows the utilization time of the generator. When the utilization time is higher than $t^{*}$, nuclear generators are economically viable. As NLDC shifts from $G^{\prime }$ to $G^{\prime \prime }$, nuclear generators with a utilization time below $t^{*}$ will be replaced by fossil energy. Area a+b represents VRE generation increase, area b+c represents nuclear power reduction, and area c−a represents the change of fossil fuel power generation. Since $G^{\prime }$ is steeper than $G^{\prime }$, area c is bigger than area a, which means that fossil fuel power generation increased, and accordingly, GHG emissions increased. Figure 1B shows the mix of capacity. See STAR Methods for a more detailed description.

The analytical framework theoretically reveals that VRECON will lead to not only a decrease in nuclear power capacity but also an increase in total GHG emissions from the electricity system. Particularly, in the process of VRECON, for every 1% increase in the VRE penetration, the nuclear power penetration will decrease by more than 1%. The analytical framework further indicates that varying VRE mix could mitigate VRECON since the VRE mix determines the shape of NLDC (Figure S1).

**Figure 2:**
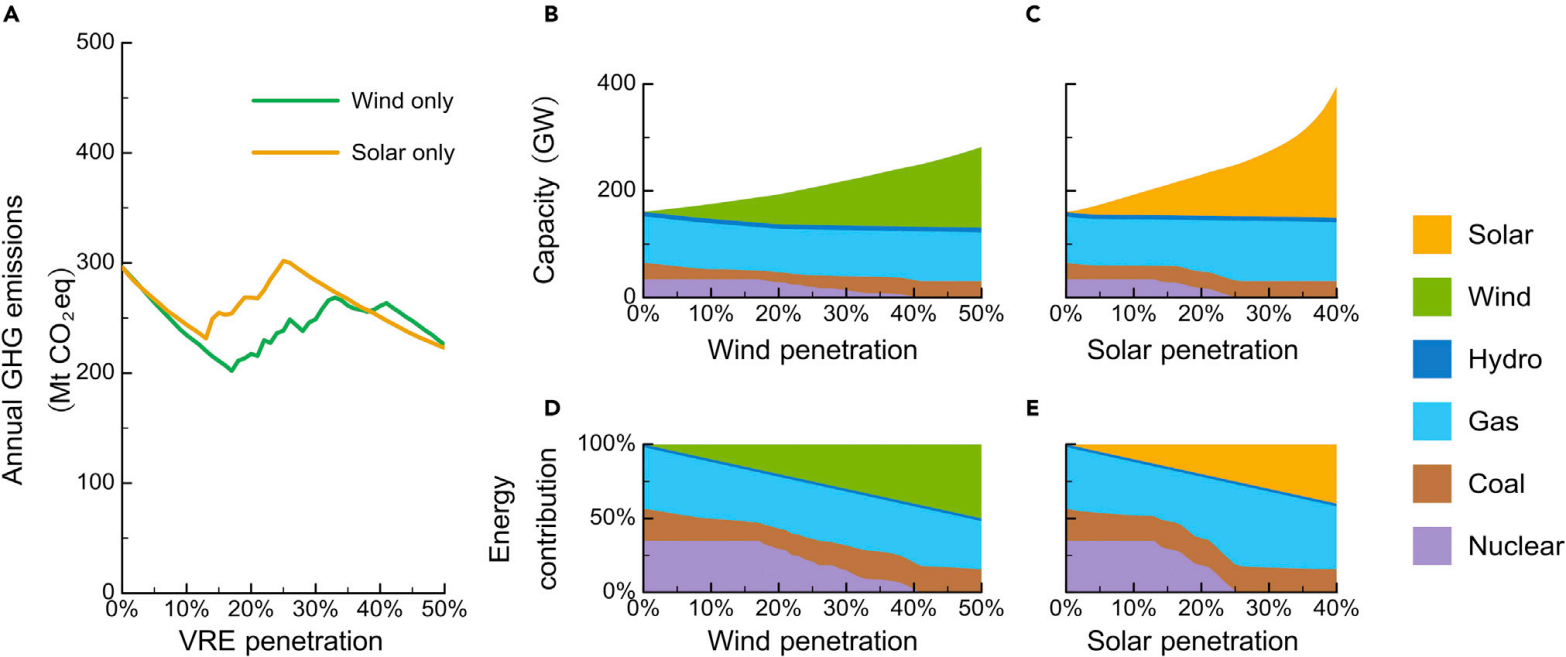
Impact of wind or solar power increase on power supply structure and GHG emissions in PJM (A) The annual GHG emissions under wind only and solar only scenarios. (B) Capacity change under wind only scenario. (C) Capacity change under solar only scenario. (D) Energy contribution change under wind only scenario. (E) Energy contribution change under wind only scenario.

**Figure 3:**
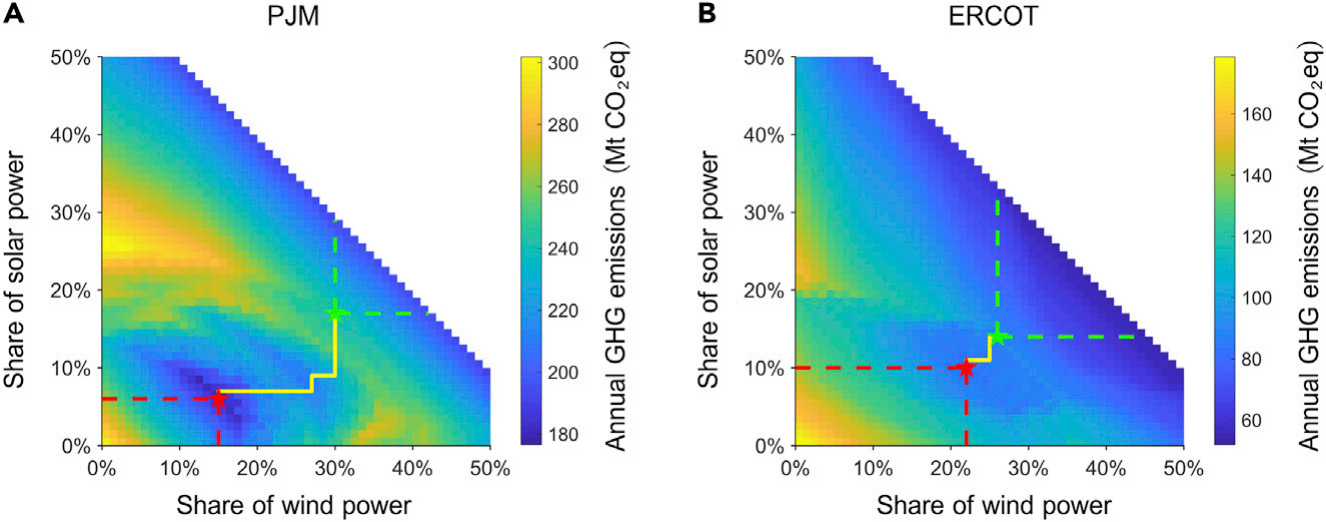
Annual GHG emissions with different VRE penetration in PJM and ERCOT The red star is the “valley point,” the yellow line is the optimal pathway to minimize GHG emission increase caused by VRECON, and the green star represents when all nuclear power is retired. On the optimal pathway, the areas enclosed by the red dotted lines indicate where the penetration of wind and solar are lower than their valley point level, and the areas enclosed by the green dotted lines indicate the wind and solar penetration that can be selected when all nuclear generators are retired. Figure S2 shows the change in nuclear power capacity. Figure S3 shows the energy contribution and capacity change on the optimal pathway.

Furthermore, we estimate quantitatively GHG emissions caused by VRECON under different VRE penetration in PJM and ERCOT by constructing the partial-equilibrium model (the detailed description of the model please see the STAR Methods). Since we focused primarily on the effects of VRE penetration increases, other parameters that might change year by year in reality, such as electricity load and fuel costs, were set to be constant in the model. The generator parameter data of coal-fired power, gas-fired power, and nuclear power is mainly collected from EPA (2020a) and NEI (2020); the hourly time-series data of electricity demand, import, export, the generation profiles of VRE, and hydropower is collected from EIA (2021a). Our results demonstrate that VRECON will push up the annual GHG emissions in both markets (Figures 2 and 3), especially in PJM where there are a lot of nuclear generators in use.

Figure 2 shows in the scenario that PJM only develops wind power (wind only scenario); VRECON will not start until wind power penetration reaches 17%. Before reaching the trigger point, VRE growth only replaces fossil energy and drives GHG decline. After wind power penetration crosses the 17% threshold, VRECON starts and boosts GHG emissions until nuclear power has been fully decommissioned. Figure 2A shows that the GHG increase during VRECON is high. When wind power penetration reaches 33%, VRECON pushes PJM GHG emissions up to a level that is even higher than when wind penetration is 5%. The expected decarbonization trajectory associated with wind power penetration is significantly distorted.

The progress of VRECON boosting GHG largely varies according to the VRE mix. For instance, VRECON starts much earlier and boosts GHG more in the solar-only scenario than in the wind-only scenario. In the solar-only scenario of PJM, VRECON will start once solar penetration reaches 13% and end when solar penetration reaches 13%. During the VRECON, every additional solar penetration causes a greater GHG boost than wind penetration. Different VRE mixes cause divergent GHG trajectories because the VRE mix determines how VRE penetration shapes the peak-off-peak differences of netload. Compared with wind power, solar power more quickly and dramatically enlarges the peak-off-peak difference, thus leading to earlier and more intense VRECON. In the solar-only scenario, every 1% more VRE penetration causes around 4.91 MtCO_2_eq emissions increase. In the wind-only scenario, every 1% more VRE penetration causes a 2.46 MtCO_2_eq increase of annual emission.

We investigated the GHG emissions associated with VRE penetration given various VRE mixes in PJM and ERCOT (Figure 3). VRECON causes the annual GHG emissions of PJM and ERCOT to increase by up to 103.0 and 32.8 MtCO_2_eq, respectively. The result also demonstrates that VRECON will inevitably cause GHG emissions to increase. No matter how the VRE mix changes, GHG emissions will increase immediately once VRE begins to crowd out nuclear generators (Figure S2). Wind power and solar power currently account for 2.9% and 0.3% of total power generation in PJM in 2019 (EIA, 2021a). However, some states in PJM set ambitious targets of VRE development, e.g., Maryland and New Jersey set their 2030 RPS target at 50%. Our analysis on PJM reflects VRECON’s occurrence and GHG-boosting effect in a market that has a very low current VRE penetration level but an ambitious goal. When PJM’s VRE market share grows from the current level to their ambitious targets, GHG emissions will decline at first but increase once VRE’s penetration triggers the VRECON. In ERCOT where the nuclear power share is relatively low, VRECON has relatively small impacts on GHG emissions. The GHG-boosting effect of VRECON will last until nuclear generators are fully retired from the market.

### Optimal VRE mix pathway minimizing the VRECON GHG-boosting effect

The generation profiles of wind and solar power are negatively correlated and lead to different degrees of VRECON (Figure 3). This leads us to explore the optimal pathway of the VRE mix that minimizes VRECON’s GHG-boosting effect. We use the yellow line in Figure 3 to represent the optimal pathways of the VRE mix. This clarifies the wind-to-solar proportion causing the mildest VRECON of every given VRE penetration level. We summarize three criteria that an optimal pathway of the VRE mix has to satisfy.

First, the optimal pathway must guarantee maximal VRE penetration before VRECON starts. We use the term "valley point" to refer to the locations with the highest VRE penetration before VRECON occurs, as shown in Figure 3. The valley point in PJM is 21% (15% for wind, 6% for solar), whereas the valley point in ERCOT is 32% (22% for wind, 10% for solar). We indicate the areas where wind and solar penetration are both below their valley point levels with red dotted lines in Figure 3. Although changing the VRE mix in the area enclosed by the red dotted line will affect GHG emissions by changing different types of fossil energy, the difference is relatively smaller than the difference caused by VRECON. Second, we find the locations with the highest VRE penetration before all nuclear power is retired, as indicated by the green star in Figure 3. Third, the optimal VRE mix pathway after the valley points must dynamically change to timely minimize GHG increase caused by VRECON and finally reaches the position of highest VRE penetration before all nuclear power is retired.

These criteria frame that VRECON will have a minimal GHG-boosting effect if VRE is integrated into the market following the optimal VRE mix pathway. The first criterion allows the electricity system to sufficiently decarbonize before VRECON starts. The second and third criteria minimize the rate of GHG increase as driven by VRECON.

The GHG trajectory associated with the pathway of optimal VRE mix calibrates the lower bound of the VRECON GHG-boosting effect in a market. The optimal VRE mix pathway is still associated with a non-negligible GHG boost due to VRECON. Even if VRE penetration follows the optimal VRE mix pathway, the expansion of VRE will enlarge the gap between peak and off-peak electricity load, thus facilitating fossil energy to gain market share from retired nuclear generators. In PJM, the share of natural gas and coal will increase from 42.2% to 51.1% when VRE penetration increases from the valley point, VRE penetration of 21%, to 47% (Figure S3). Correspondingly, annual GHG emissions in PJM will rebound from 176.5 to 241.1 MtCO_2_eq. As an intuitive comparison, the quantity of this rebound is equivalent to 1.3 times the total GHG emissions in Denmark in 2018 (49.7 MtCO_2_eq), a country ranked 37th in the world in terms of gross national product (OECD, 2021; IMF 2021). In ERCOT, following the optimal pathway, all nuclear generators will be retired when VRE penetration reaches 40%. The corresponding annual GHG emissions will rebound to 88.2 from 82.9 MtCO_2_eq.

### Refining the design of RPS for GHG abatement

Our research shows that considering VRECON and defining VRE mix requirements will be important to ensure the effectiveness of RPS in climate change mitigation. However, some regions do not establish carve-outs within their RPS to control the VRE mix (National Conference of State Legislatures, 2020). Even in those regions where carve-outs have been established, they are primarily motivated by promoting diversity in VRE rather than avoiding VRECON, which may lead to inappropriate outcomes. In PJM, to integrate the largest VRE before VRECON occurs, wind and solar power should account for 15% and 6% of the total power generation, respectively. Yet, wind power is superior to solar power, both in terms of current realities (10:1 in 2019) and carve-outs (National Conference of State Legislatures, 2020).

**Figure 4:**
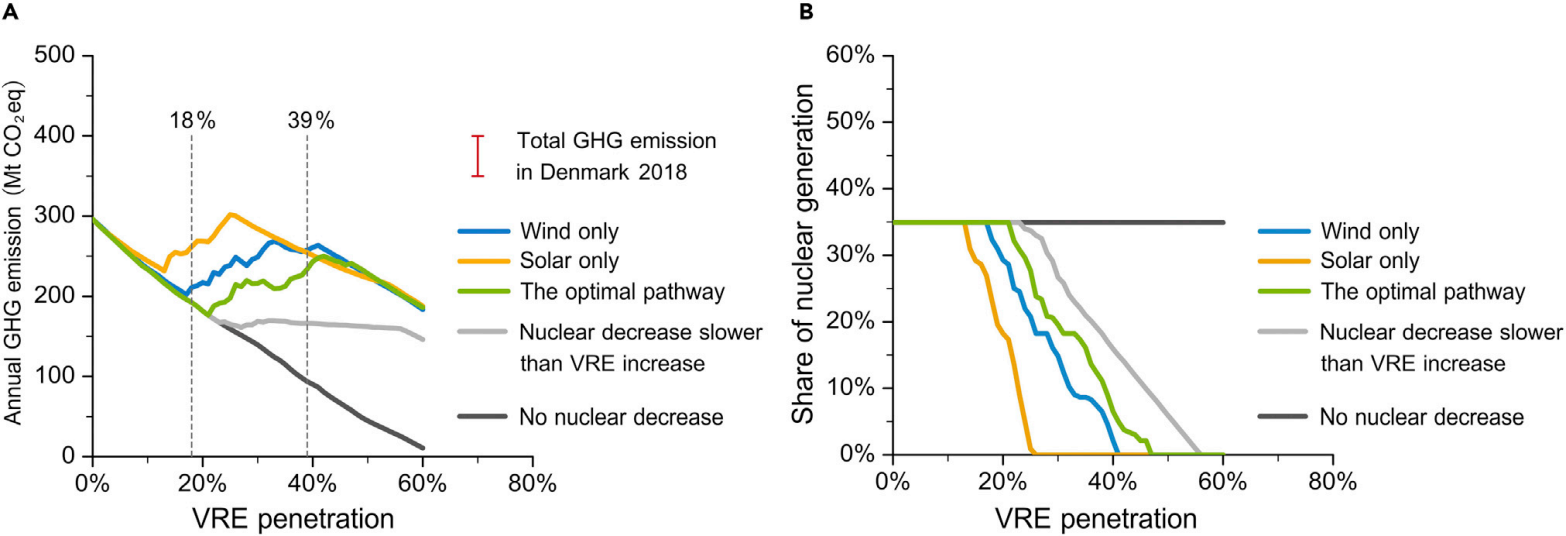
Annual GHG emissions and nuclear power share on different VRE mix pathways and under nuclear-protection policy scenarios in PJM (A) Annual GHG emissions. (B) Nuclear power share. Before and after VRECON occurs, the change of the VRE mix will also lead to a slight GHG emission difference. For simplicity, the “optimal pathway” line depicts the pathway with the lowest emissions in the areas enclosed by the red and green dotted lines of Figure 3.

The absence of a VRE mix requirement or an unsuitable VRE mix results in a significant risk of GHG abatement because of the GHG-boosting effect of VRECON. GHG emissions in PJM will be largely uncertain if the VRE mix requirement is absent. Figure 4A shows the highly divergent GHG trajectories due to the same pace of VRE-penetration progress. Even following the optimal VRE mix pathway, VRECON may largely increase PJM annual GHG emissions. Compared with the optimal pathway, the wind-only pathway will cause 18.3–59.0 MtCO_2_eq of additional GHG emissions each year during the VRECON process, when VRE penetration is between 18% and 39%, which is 0.37–1.19 times of the total GHG emissions in Denmark in 2018. The solar-only scenario will result in higher GHG emissions. Compared with the optimal pathway, the solar-only pathway will bring 103.9 MtCO_2_eq additional GHG emissions per year when VRE penetration reaches 25%, which is 2.1 times the total GHG emissions in Denmark. Even in the markets with low penetration of nuclear power energy, it is also necessary to set the VRE mix requirement associated with their RPS. In ERCOT, when the VRE penetration is between 29% and 39%, the wind-only and solar-only pathways would lead to 14.1–33.6 MtCO_2_eq additional GHG emissions each year compared with the ERCOT optimal pathway.

Combined with the potential impact of VRECON on GHG emissions, it is imperative to identify the optimal pathway and refine the design of RPS. It is worth noting that different electricity markets have different optimal pathways. This is because the optimal pathway is affected by the load, the VRE output curves, and other factors. And the difference in resource endowment may render the electricity market overly reliant on a single type of VRE. As mentioned above, PJM may rely too much on wind power to achieve RPS, leading to VRECON starting earlier.

### Rethinking the time point of nuclear power retirement

No matter how the VRE mix is adjusted, the potential risk of the VRECON GHG-boosting effect cannot be completely avoided. However, it is possible to minimize VRECON by controlling the time point of nuclear power retirement, i.e., avoiding nuclear power early retirements. The decrease of nuclear power generation driven by VRECON is always faster than the increase of VRE penetration. Take PJM as an example. Even following the optimal pathway, VRECON occurs when VRE penetration increases from 21% to 47%, leading to the decrease of nuclear power share from 34.9% to 0 ( Figure 4B). This is because the increase in VRE has made more flexible power generation necessary.

To illustrate the emission reduction role of nuclear power in the process of pursuing VRE development goals, we control the decline of nuclear power generation at a slower rate through specific policies as shown in Figure 4B. If we set the minimum ratio of nuclear power exogenously, assuming that after VRECON occurs, the decreasing share of nuclear power should not be greater than the increasing share of VRE (“nuclear decrease slower than VRE increase” in Figure 4B). The GHG decrease driven by the VRE increase is similar to the GHG increase caused by VRECON. When VRE penetration reaches 47%, the annual GHG emissions are 163.3 MtCO_2_eq under the scenario of “nuclear decrease equal to VRE increase,” which is 68% of the scenario with no nuclear decrease limitation (241.1MtCO_2_eq). By limiting the decrease of nuclear power generation, the GHG-boosting effect is mitigated. If the decrease in nuclear power generation is banned ("no nuclear decrease" in Figure 4A), VRECON will not occur.

The analyses above suggest policymakers should rethink the retirement policies of nuclear power during the decarbonization progress. The time point of nuclear retirement plays a critical role as the electricity system transitions to zero-carbon in the future. To ensure the GHG abatement effect of RPS, policymakers need to stress the coordinated development between VRE and nuclear power.

## Discussion

In the above analysis, nuclear power output is assumed to be completely inflexible. However, with technological advances, the flexible output of nuclear power likely will be realized. Some studies suggest that improving the flexibility of nuclear generators would save variable operation and maintenance (O&M) costs and thus increase their profitability in the future with a high VRE share (Cany et al., 2018; Jenkins et al., 2018; Kopiske et al., 2017; Loisel et al., 2018; Lykidi and Gourdel, 2015, 2017). We conducted a sensitivity analysis on nuclear power output flexibility (see STAR Methods). The results show that making nuclear power more flexible allows VRECON to occur later and reduce GHG emissions. The cost-effectiveness of converting existing nuclear generators to flexible power sources after taking into account VRECON needs to be studied further.

Furthermore, it is also necessary to examine how VRECON is impacted by energy storage and demand-side management (DSM). Energy storage equipment transfers power generation from off-peak hours to peak hours to mitigate the impact of VRE intermittency (Dunn et al., 2011; Ibrahim et al., 2008; Nyamdash et al., 2010). DSM promotes the transfer of electricity demand from peak hours to off-peak hours (Pina et al., 2012; Strbac, 2008). Currently, there are still different opinions on whether energy storage and DSM will reduce or increase GHG emissions (Baumgärtner et al., 2019; Carson and Novan, 2013; Cooper et al., 2014; Hittinger and Azevedo, 2015; Lin et al., 2016; Linn and Shih, 2019). Our research provides a new perspective on these debates. Since VRECON is caused by the intermittency of VRE, and energy storage and DSM can address this problem, energy storage and DSM may help reduce GHG emissions in the electricity system by mitigating VRECON. In addition, adjusting hydropower operation also helps improve electricity system flexibility and addresses the intermittency of wind and solar power. Studies have found that smart management of present and future hydropower plants can support substantial grid integration of solar and wind power, mitigating intermittency and limiting natural gas consumption (Engeland et al., 2017; Sterl et al., 2020, 2021). This suggests that adjusting hydropower operation may help alleviate VRECON.

### Conclusion

Our research provides a counter-intuitive phenomenon that VRE could have a negative impact on electricity system GHG emissions—the potential risk that VRE crowding out nuclear power will boost GHG emissions. This is because the intermittent nature of wind and solar power prevents them from fully occupying the market share left by retired nuclear power, thus encouraging the expansion of fossil fuel power generation. Since nuclear power currently accounts for 10.1% of global electricity generation (IEA, 2021), it is urgent to conduct more comprehensive research on the issue and examine the impact of VRECON on climate change mitigation progress worldwide.

Through constructing a partial equilibrium model, we estimated quantitatively the impact of VRECON on GHG emissions in PJM and ERCOT in the United States. We found VRECON intensely increases PJM annual GHG emissions. Even though the VRE mix is in the best position to mitigate VRECON, it still has the chance to lead to an increase in annual GHG emissions of 73.6 MtCO_2_eq.

To ensure future GHG emission reductions, policymakers need to consider VRECON when formulating energy transition policies. Some researchers suggest that technology-specific VRE requirements, such as solar carve-outs in RPS, would not yield additional GHG emissions mitigation (Fell and Johnson, 2020; Novacheck and Johnson, 2015). However, our research reveals the significance of such requirements in mitigating VRECON and reducing GHG emissions. Considering VRECON, it is critical to explore VRE mix requirements in RPS that balance economic and environmental benefits. For example, our research shows that VRECON can be mitigated to the greatest extent by keeping wind and solar generation in PJM at about 2:1. Furthermore, VRECON will encourage policymakers to re-examine some of the policy options in energy transitions. For example, should policymakers adopt policies that support only renewable energy, e.g., RPS and renewable energy subsidies, or adopt policies that support all low-carbon power sources, e.g., carbon taxes and clean energy standards? Would these trade-offs leave the door open for nuclear power? Policies such as postponing the decline in nuclear power’s share, and encouraging technologies that increase the flexibility of the electricity system, should also be considered in a future with high VRE penetration. Regardless, more research is warranted on how to promote VRE development by considering the system constraints of an electricity market to realize global climate change mitigation goals.

### Limitations of the study

This paper reveals that VRE could have a negative impact on electricity system GHG emissions owing to crowding out nuclear power. Nevertheless, there are several limitations to the present study and they deserve exploration in the future. First, we only analyzed VRECON caused by the volatility of VRE. In reality, VRECON could be more serious than our estimation. For instance, our model does not consider VRE’s uncertain nature. The uncertainty of VRE generation usually increases the demand for ancillary services, such as spinning reserves. Yet, nuclear generators have weaker competitiveness in providing ancillary services than do fossil fuel generators. Consequently, the uncertainty of VRE could exacerbate VRECON GHG-boosting effect.

Second, our current policy discussion only addresses parts of the issue about refining the current renewable and low-carbon policies. Two directions of policy discussions deserve further study. One is the optimal resolution of the relative policies. For instance, our research only considers two stylized VREs, wind and solar power, whereas VRE can be subdivided into additional types, e.g., onshore and offshore wind power. Each of these technologies has different generation characteristics and may result in varying extents of VRECON, which warrants further investigation. The other direction is to explore the policies of coordinating other technologies, such as energy storage and low-carbon energy besides VRE.

Third, this paper does not sufficiently analyze the cost-effectiveness of candidate policies of mitigating the VRECON GHG-boosting effect. Our analysis pointed out that the VRECON GHG-boosting effect could be managed by both the policies of optimizing the VRE mix and controlling the pace of nuclear retirement. In addition, there may be more candidate policies, as discussed in the previous paragraph. In future research, it is necessary to examine and compare the economic burdens of these candidate policies.

## STAR★Methods

### Key resources table

**Table:** 

REAGENT or RESOURCE	SOURCE	IDENTIFIER
**Deposited Data**		

Hourly electricity demand, import, and export of PJM and ERCOT	EIA	https://www.eia.gov/electricity/gridmonitor/index.php
Heat rates and capacities of existing generators in PJM and ERCOT	EPA	https://www.epa.gov/energy/emissions-generation-resource-integrated-database-egrid

**Software and Algorithms**		

MATLAB R2018b	MathWorks	https://matlab.mathworks.com/
YALMIP	Löfberg, 2004	https://yalmip.github.io/
GUROBI	Gurobi	https://www.gurobi.com/

### Resource availability

Further information and requests for resources should be directed to and will be fulfilled by the lead contact, Yang Yu (yangyu1@tsinghua.edu.cn).

#### Materials availability

The study did not generate new materials.

### Method details

#### Analytical framework

We propose an analytical framework for understanding how variable renewable energy crowds out nuclear power. The analytical framework used in this article, also known as the "screening curves" approach. Refer to to the literature for the introduction to the “screening curves” approach (Biggar and Hesamzadeh, 2014; Stoft, 2002).

In this analytical framework, the flexibility constraints of the generators are all ignored. To obtain the optimal dispatch plan, generators are scheduled according to the order of marginal cost from the lowest to the highest (the merit order). To solve the market equilibrium through the "screening curves" approach, we need to construct the load duration curve (LDC) and net load duration curve (NLDC). Net load is the difference between load and renewable energy output. A load duration curve or residual load-duration curve can be constructed by measuring the total load or residual load at hourly intervals for each of the 8760 h in a year, sorting them, and graphing them starting with the highest load. The result is a curve that slopes downward from the maximum load in the peak hour, hour 1, to the minimum load, baseload, in the most off-peak hour, hour 8760 (Stoft, 2002). In general, the high penetration of renewable energy tends to make the NLDC steeper (Ueckerdt et al., 2013).

Consider a simplified market with only one type of existing nuclear technology and one type of fossil fuel technology. For simplicity, we will not distinguish between new and existing fossil power generators. Figure S4 shows the relationship between the generation cost and utilization time of a 1 MW generator in a year. The intercept of the curve represents the fixed cost, and the slope represents the marginal cost. Compared with fossil fuel, nuclear power generation has a relatively high fixed cost and low marginal cost. When the utilization time is lower than $t^{*}$, nuclear generators will be replaced by fossil fuel generators because of their poor economy. Assume that the capacity of the existing nuclear power is NC.

When there is no VRE in the market, the LDC G is shown in Figure S4C. Figure S4C also shows the electricity generation from fossil fuel and nuclear power.

Considering whether the VRE increase would crowd out nuclear power, the process could be divided into three stages. First, the increase in VRE led to NLDC $G^{\prime }$ and it has not yet crowded out nuclear power (Figures S4D and S4E). Figure S4E shows the parts of fossil energy and nuclear power replaced by VRE.

Second, VRE crowd out nuclear power. Consider a case where the NLDC $G^{\prime }$ makes the utilization time of the power generator with the lowest utilization time exactly equal to $t^{*}$, as Figure S4G shows. A further increase in VRE led to NLDC $G^{\prime \prime }$. From NLDC $G^{\prime }$ to NLDC $G^{\prime \prime }$, the electricity generated by fossil fuel and nuclear power replaced by VRE can be represented by area a and area b, respectively. In addition, some nuclear generators have been replaced by fossil fuels due to low utilization time (Figure S4F). This effect leads to the substitution of nuclear power generation by fossil power generation and can be represented by area c. In the above process, the change in fossil energy generation can be expressed as area c minus area a. As shown in Figure S1, the increase in VRE will make the NLDC steeper. Therefore, $G^{\prime \prime }$ is steeper than $G^{\prime }$, area c is bigger than area a, which means that fossil fuel power generation increased, and accordingly, GHG emissions increased.

Third, when all nuclear power is retired, there will be only fossil fuel and VRE in the market, and the increase in renewable energy will reduce GHG emissions.

Furthermore, as Figure S1 shows, the proper combination of wind power and solar power can lead to a relatively flat NLDC, as opposed to only wind or only solar. This is because the time distribution of wind power generation and solar power generation is negatively correlated, which means that the proper combination of wind and solar power could mitigate VRECON.

#### The numerical model

We build a numerical model to estimate the impact of increasing VRE on the electricity generation mix and GHG emissions in the electricity market. We consider the midterm (or medium run), a time frame of 10–20 years. In the midterm, the model takes into account existing generators and allows for retirement and investment. For the detailed definition of midterm, please see Hirth (2013). This numerical model is a partial equilibrium model of the electricity market. There are two basic assumptions in the model: (1) the electricity market is perfectly competitive, (2) the electricity load is inelastic and has no uncertainty. Therefore, the model is implemented as a mixed-integer linear program, which solves the investment, retirement, and dispatch plan that minimizes the electricity supply cost as the market equilibrium result. We model the optimization problem by using the YALMIP package (Löfberg, 2004) and solve it with GUROBI.

The model contains six types of power sources: coal, natural gas, nuclear, hydropower, wind power, and solar power. Among them, coal is subdivided into bituminous, subbituminous, and lignite. According to technical characteristics, natural gas generators are divided into combined cycle, combustion turbine, and steam turbine. Nuclear generators are divided into pressurized water reactors and boiling water reactors.

#### Data

The input data of the numerical model can be divided into two parts, time-series data and generator parameters.

Time series data includes the electricity demand, the electricity import, the electricity export, the generation profiles of VRE, and the generation profiles of hydropower. Historical data in 2019 is selected. All the time series data comes from EIA, 2021a. The electricity demand, import, and export are set to be consistent with historical data in the typical weeks and remain constant. For simplicity, the electricity load in the following refers to electricity demand minus electricity import and plus electricity export, which is the total output of all generators in this market should be reached. We determine the VRE profiles by referring to the historical output curve and the average capacity factors of wind power and solar power in 2019. The historical total VRE output curves are scaled down and used as the profiles of the 1MW generators (35.4% for wind power and 24.9% for solar power (Statista, 2021)).

Referring to the study of de Sisternes and Webster (2009), we choose four typical weeks and one typical day with the highest load to represent the annual load distribution. The load curves and VRE profiles of typical weeks and typical days are shown in Figures S5 and S6. Figures S7 and S8 show the approximations of LDC and NLDC.

The sources of generator parameters are as follow. The value of generator parameters is shown in Tables S1 and S2. For existing fossil fuel generators (coal and natural gas), we group them according to their heat rates (Btu/kWh) by a clustering method and get the approximations (Figures S9 and S10). In terms of fossil fuel costs, we use the average value of monthly fossil fuel costs of 2019 as the input parameter (EIA, 2021b).

**Table:** The sources of generator parameters

Parameters		References
Fossil fuel	Coal and natural gas costs	(EIA, 2021b)
	Existing capacity	(EPA, 2020a)
	Heat rates of existing generators	(EPA, 2020a)
	Heat rates of new generators	(EIA, 2016)
	Start-up costs	(Schill et al., 2017)
	Minimum output	(Schill et al., 2017)
	O&M costs	(EIA, 2016; Schröder et al., 2013)
	Overnight capital cost	(EIA, 2016)
	Lifetime	(Schröder et al., 2013)
	GHG emission factors	(EPA, 2020b)
VRE	O&M costs	(EIA, 2016)
	Overnight capital cost	(NREL, 2019)
	Lifetime	(Schröder et al., 2013)
Nuclear	List of existing generators	(EPA, 2020a)
	Costs	(NEI, 2020)
	Net capacity	(EIA, 2021c)
Other	Interest rate	(NREL, 2019)
	Global warming potential of GHG	(Pachauri et al., 2007)

Investment costs refer to to the amount of revenue the generator owner needs to receive from the electricity market each year to offset the costs of installation. Investment costs IC can be described as Equation 1. Where OC refers to the overnight capital cost, i refers to the interest rate, and N refers to the lifetime. The interest rate is set at 5.4% (NREL, 2019).

(Equation 1) $$\begin{array}{l} IC=OC\frac{i\left(1+i\right)N}{{\left(1+i\right)}^{N}-1} \end{array}$$

#### The optimization problem

To quantify the impact of VRECON on GHG emissions and to further explore the extent to which VRECON can be mitigated by the VRE mix, we need to simulate a large number of situations in an electricity market. In this study, we investigate the process of VRE penetration rising from 0 to 60%, and the penetration of wind power and solar power both change by 1% step by step. Accordingly, we need to calculate the results of 1891 different situations. We built this model which contains the most important constraints in electricity market simulation, such as power balance and capacity limits. It is suitable for calculating a large number of simulations, and we use it to estimate the capacity expansion, power generation share, and GHG emission of the electricity market under the assumption of perfect competition. Some detailed constraints are not considered, such as ramp rates, minimum uptime, and minimum offtime. Not considering these constraints may cause the model to underestimate VRECON. This is because, if we do not consider these constraints of fossil fuel power generation, the fossil fuel power generation will be more flexible than it should be; thus, with increasing VRE penetration, more fossil fuel power generation will replace nuclear power to obtain enough flexibility to match VRE. Such underestimation does not affect our main conclusion that VRECON causes GHG emissions increase. In addition, this simplified method is often used to study the issues related to electricity systems (Deetjen and Azevedo, 2019; Fell and Linn, 2013; Linn and Shih, 2019). The lists of sets, variables, and parameters are as follow.

**Table:** List of sets, variables, and parameters in the numerical model.

Sets	Description	Unit
g∈G	Set of GHG	–
i∈I	Set of fossil fuel generator groups	–
j∈J	Set of VRE	–
k∈K	Set of nuclear generators	–
l∈L	Hydro power and geothermal power	–
t∈T	Set of hours of various typical weeks/day	–
w∈W	Set of typical weeks and typical day. w=1,2,3,4 representing the typical week. w=5 representing the typical day	–
existing	Indicate existing generators	–
new	Indicate new generators	–

Continuous variables	Description	Unit

$fadd_{i}$	Newly invested capacities of fossil fuel generator groups	MW
$fcap_{i}$	Available capacities of fossil fuel generator groups	MW
$fol_{w,t,i}$	Hourly online capacities of fossil fuel generator groups	MW
$fp_{w,t,i}$	Hourly power generation of fossil fuel generator groups	MWh
$fret_{i}$	Retired capacities of fossil fuel generator groups	MW
$fup_{w,t,i}$	Total capacities of fossil fuel generators in group i started up in typical weeks/day w hour t	MW
ghge	Total GHG emissions	
$np_{w,t,k}$	Hourly power generation of nuclear generators	MWh
$radd_{j}$	Newly invested capacities of VRE	MW
$rp_{w,t,j}$	Hourly power generation of VRE	MWh
$rret_{i}$	Retired capacities of VRE	MW
totalcost	Total costs of electricity supply	$

Binary variables	Description	Unit

$nu_{k}$	Decision variable of nuclear generators (1 if continue to serve, 0 if retire)	1 of 0

Parameters	Description	Unit

$D_{w,t}$	Electricity load	MW
$FCC_{i}$	Investment costs of fossil fuel generators	$/MW
$FE_{i,g}$	GHG emission factors of fossil fuel power generation	g/MWh
$FEXI_{i}$	Existing capacities of fossil fuel generator groups	MW
$FFC_{i}$	Fuel costs of fossil fuel power generation	$/MWh
$FFOM_{i}$	Annual fixed operation and maintenance costs of fossil fuel generators	$/MW
$FMIN_{i}$	The ratio of minimum outputs to nameplate capacities of fossil fuel generators	–
$FSC_{i}$	Start-up costs of fossil fuel generators	$/MW
$FVOM_{i}$	Variable operation and maintenance costs of fossil fuel power generation	$/MWh
$GWP_{g}$	100-year global warming potential compared to carbon dioxide	gCO_2_eq/g
$H_{w}$	The scaling factor of typical weeks/day	–
i	Interest rate	–
IC	Investment costs	$
N	Lifetime	year
$NC_{k}$	Costs of nuclear power generation	$/MWh
$NCAP_{k}$	Net capacity of nuclear generators	MW
$NMIN_{k}$	The ratio of minimum stable outputs to nameplate capacities of nuclear generators	–
NPL	Limitations on nuclear power generation	–
OC	Overnight capital cost	$/kW
$OP_{w,t,l}$	Hourly generation of hydro power	MWh
$RA_{w,t,j}$	Hourly availability of VRE	–
$RCC_{j}$	Investment costs of VRE	$/MW
$REXI_{j}$	Existing capacities of VRE	MW
$RFOM_{j}$	Annual fixed operation and maintenance costs of VRE	$/MW
$RPS_{j}$	Renewable portfolio standards for different type of technologies	–

#### Objective function

Based on the assumption of perfect competition and inelasticity of electricity demand, the model takes the solution of minimizing the generation cost as the result of market equilibrium. As Equation 2, generation costs include fuel costs, variable operation and maintenance (O&M) costs, fixed O&M costs, start-up costs, and investment costs. In reality, the start-up costs are affected by offtime. As modeling offtime-dependent start-up costs is computationally demanding, we use a simplified approach, according to Schill et al. (2017): coal generators are assumed to carry out warm starts with 50% of the typical cold-start fuel requirement, natural gas generators are assumed to typically carry out cold starts, incurring 100% of the start-up fuel requirements presented in the Table S1.

(Equation 2) $$\begin{array}{l} total\;\cos\;t=\sum_{w,t,i}H_{w}(fp_{w,t,i}FFC_{i}+fp_{w,t,i}FVOM_{i}+fup_{w,t,i}FSC_{i}\\ +\sum_{w,t,k}H_{w}NCAP_{k}NC_{k}nu_{k}\\ +\sum_{i}cap_{i}FFOM_{i}+\sum_{j}rcap_{j}RFOM_{j}\\ \begin{array}{l} +\sum_{i}fadd_{i}FCC_{i}+\sum_{j}radd_{j}RCC_{j} \end{array} \end{array}$$

#### Energy balance

The dynamic equilibrium of the electricity system is the most basic constraint in this model, which requires the sum of the electricity generation to be equal to the electricity load at all hours, as Equation 3. $D_{w,t}$ refers to hourly electricity demand minus electricity import and plus electricity export.

(Equation 3) $$\begin{array}{l} \sum_{i}fp_{w,t,i}+\sum_{j}rp_{w,t,j}+\sum_{k}np_{w,t,k}+\sum_{h}OP_{w,t,h}=D_{w,t}\;\forall w,t \end{array}$$

#### Generation

Constraint (4) indicates that at any time, the output of the fossil energy is less than its online capacity and greater than its minimum stable output level. Constraint (5) limits the online capacity to be greater than zero and less than the total existing capacity. Constraints (6–7) describe the start-up capacity at each hour.

(Equation 4) $$\begin{array}{l} fol_{w,t,i}FMIN_{i}⩽fp_{w,t,i}⩽fol_{w,t,i}\;\forall w,t,i \end{array}$$

(Equation 5) $$\begin{array}{l} 0⩽fol_{w,t,i}⩽fcap_{i}\;\forall w,t,i \end{array}$$

(Equation 6) $$\begin{array}{l} fol_{w,t+1,i}⩽fol_{w,t,i}+fup_{w,t+1,i}\;\forall w,t,i \end{array}$$

(Equation 7) $$\begin{array}{l} fup_{w,t,i}⩾0\;\forall w,t,i \end{array}$$

As Equation 8, the outputs of VRE are constrained by their profiles. Wind power and solar power can be curtailed. Hence, with increasing VRE, nuclear power will not retire because the energy balance constraints cannot be met.

(Equation 8) $$\begin{array}{l} 0⩽rp_{w,t,j}⩽rcap_{j}RA_{w,t,j}\;\forall w,t,j \end{array}$$

In all analyses except Supplemental analysis of Nuclear power flexibility, we use constraint (9) to model the generation of nuclear power. Unless a nuclear generator retires, its output must be maintained at its net capacity. In the supplementary analysis of nuclear power flexibility (see Method Details 3.3 Nuclear power flexibility), we use constraint (10) instead of constraint (9), which means that the nuclear generator can reduce its output.

(Equation 9) $$\begin{array}{l} np_{w,t,k}=nu_{k}NCAP_{k}\;\forall w,t,k \end{array}$$

(Equation 10) $$\begin{array}{l} nu_{k}NCAP_{k}NMIN_{k}⩽np_{w,t,k}⩽nu_{k}NCAP_{k}\;\forall w,t,k \end{array}$$

#### Capacity

In the medium-term market equilibrium, the model makes endogenous decisions about investment and retirement based on existing capacity. Constraints (11–17) are the logical constraint of capacity.

(Equation 11) $$\begin{array}{l} fadd_{i}=0\;\forall i\in existing \end{array}$$

(Equation 12) $$\begin{array}{l} fadd_{i}⩾0\;\forall i\in new \end{array}$$

(Equation 13) $$\begin{array}{l} radd_{j}⩾0\;\forall j \end{array}$$

(Equation 14) $$\begin{array}{l} 0⩽fret_{i}⩽FEXI_{i}\;\forall i \end{array}$$

(Equation 15) $$\begin{array}{l} 0⩽rret_{j}⩽REXI_{j}\;\forall j \end{array}$$

(Equation 16) $$\begin{array}{l} fcap_{i}=FEXI_{i}+fadd_{i}-fret_{i}\;\forall i \end{array}$$

(Equation 17) $$\begin{array}{l} rcap_{j}=REXI_{j}+radd_{j}-rret_{j}\;\forall j \end{array}$$

#### Greenhouse gas emissions

Our estimates include the three categories of major GHG emitted during electricity generation: carbon dioxide (CO_2_) methane (CH_4_), and nitrous oxide (N_2_O). We estimate the GHG emission by Equation 18. Where $FE_{i,g}$ represents the emission factor (g/MWh) of gas type g and fossil fuel generator groups i, which is estimated by the GHG emission factors of fossil fuel combustion (g/MMBtu) and generators' heat rates (MMBtu/MWh) (EPA, 2020a; 2020b). The GHG emission factors and global warming potentials GWP (Pachauri et al., 2007) are shown in Table S3. In this research, we ignore the GHG emissions from VRE, hydropower, and nuclear power

(Equation 18) $$\begin{array}{l} ghge=\sum_{w,t,i,g}H_{w}fp_{w,t,i}FE_{i,g}GWP_{g} \end{array}$$

#### Renewable portfolio standards

Since we assume that the electricity load, electricity import, and electricity export are exogenous, we describe RPS with constraints limiting the proportion of each type of VRE in the total electricity generation by referring to relevant literature (Fell and Linn, 2013), as constraint (19).

(Equation 19) $$\sum_{w,t}H_{w}rp_{w,t,j}⩾RPS_{j}\sum_{w,t}H_{w}D_{w,t}\;\forall j$$

To estimate the impact of the gradual increase in RPS targets on GHG emissions, we calculated the market equilibrium results for a series of different RPS targets, namely, a series of optimization problems with different parameters in RPS constraints.

#### Limitations on nuclear power retirement

In the analysis of limiting the decline of nuclear power generation, we add constraint (20) to the optimization problem. The share of nuclear power is set to a minimum exogenously NPL.

(Equation 20) $$\begin{array}{l} \sum_{w,t,k}H_{w}np_{w,t,k}⩾NPL\sum_{w,t}H_{w}D_{w,t} \end{array}$$

#### Model validation

Supporting the validity of this model, Figure S11 compares observed outcomes in PJM and ERCOT in 2019 with outcomes if we simulate our model for the year 2019 and do not allow investment or retirement. Here, the outputs of VRE are set to be consistent with historical data in typical weeks (Figures S5 and S6).

In general, the simulation results are close to the actual observation outcomes, which shows that the typical weeks we selected can properly represent the whole year. The share of nuclear power generation in the model is higher than the observed outcomes, which may be because we ignore the overhaul and unexpected shut down of nuclear generators. The model estimates a higher proportion of electricity generated from fossil fuels than observed outcomes, possibly because we ignore other types of electricity generation, such as biomass and oil.

#### Supplemental analysis

Three sets of supplemental analyses were performed on the model of PJM to assess the impact of the input parameters on the results. There is a higher nuclear power share in PJM than that in ERCOT, which will lead to PJM suffering more in the GHG emission increase caused by VRECON. Hence, we take PJM as an example. In general, changes in these input parameters will affect the results of the numerical model, but will not change our conclusions.

#### Natural gas cost

The natural gas cost varies greatly in different years (EIA, 2021b) and has a great influence on the electricity market. To improve the credibility of our results, we make a supplemental analysis for the cost change of natural gas in PJM. Assume that the cost of natural gas increases and decreases by 10% compared to the average cost in the U.S. power sector in 2019 (2.94 $/MMBtu), the results are shown in Figure S12. It is found that low natural gas cost will reduce GHG emissions in the power system and vice versa. But VRECON’|'s impact on GHG emissions is similar under different natural gas cost scenarios.

#### Nuclear power cost

Since the cost of nuclear power in the United States has shown a downward trend in the past five years (NEI, 2020), we have analyzed the situation where the cost of nuclear power has dropped by 10% or 20%. The result is shown in Figure S13. The fall of nuclear power cost will delay VRECON, but VRECON will still cause an increase in GHG. Since the investment of nuclear generators is not considered in our model, before VRECON happens, the change in the costs of nuclear power will not affect GHG emissions.

#### Nuclear power flexibility

In the previous analysis of this research, we assume that the output of nuclear power is always equal to its capacity since these nuclear generators have no precedent for flexible operation of adjusting output between hours. In this supplementary analysis of nuclear power flexibility, we replace constraint (9) with constraint (10) which means that the output of nuclear generators can be reduced to 75% or 50% of its capacity. With reference to Jenkins et al. (2018), we assume that nuclear power can save variable O&M costs (0.5 $/MWh) by reducing output, but cannot save fuel costs. The analysis here is intended only to indicate the potential of nuclear flexibility to mitigate VRECON. We did not take into account the additional costs of converting existing nuclear generators to flexible power sources, such as the cost of equipment modifications and additional maintenance. The result is shown in Figure S14. Making nuclear power more flexible would allow VRECON to happen later and help reduce GHG emissions.

## Data Availability

• This paper analyzes existing, publicly available data. These accession numbers for the datasets are listed in the key resources table and Method Details. • This paper does not report original code, which is available for academic purposes from the lead contact upon reasonable request. • Any additional information required to reanalyze the data reported in this paper is available from the lead contact upon request.
