# Supplementary material for: Potential greenhouse gas risk led by renewable energy crowding out nuclear power
Source: iScience. 2022 Jan 7;25(2):103741. doi: 10.1016/j.isci.2022.103741 (PMC8814763; doi:10.1016/j.isci.2022.103741)
Supplement: Document S1. Figures S1–S14 and Tables S1–S3 [file mmc1.pdf]

**iScience, Volume *25***

## Supplemental information

# Potential greenhouse gas risk led by renewable energy crowding out nuclear power

**Xiaoli Zhao, Zewei Zhong, Xi Lu, and Yang Yu**

## Supplemental Figures

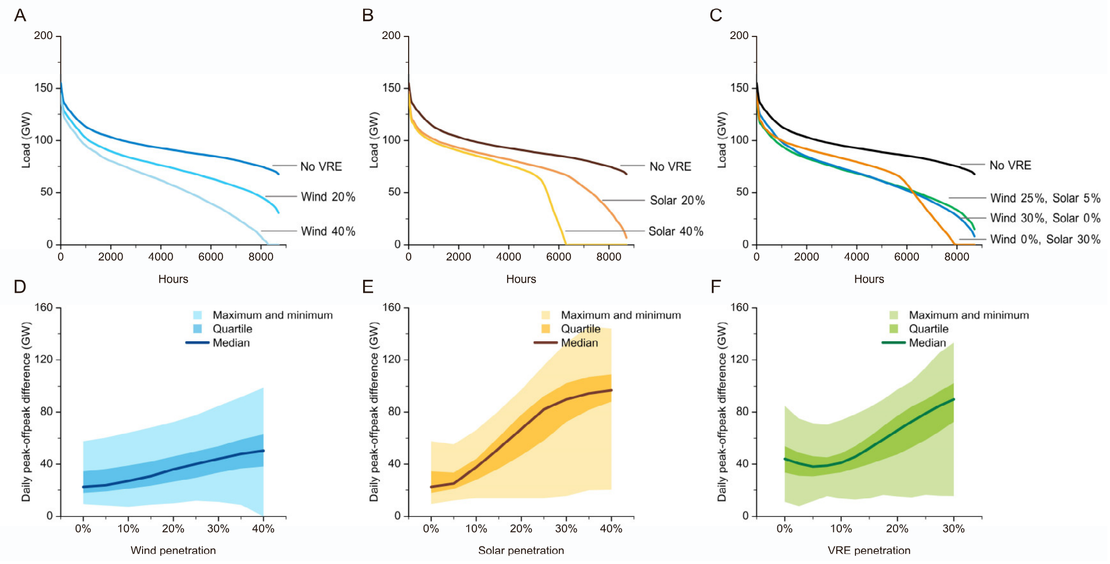


**Figure S1. Influence of VRE on net load volatility in PJM, related to STAR Methods**

(A-C) Load duration curves and net load duration curves.

(D-F) Daily peak-offpeak difference.

The (net) load duration curves show all individual (net) load hours in a subsequent order. We estimate the net load based on VRE power generation and load data in 2019.

LDC-load duration curve.

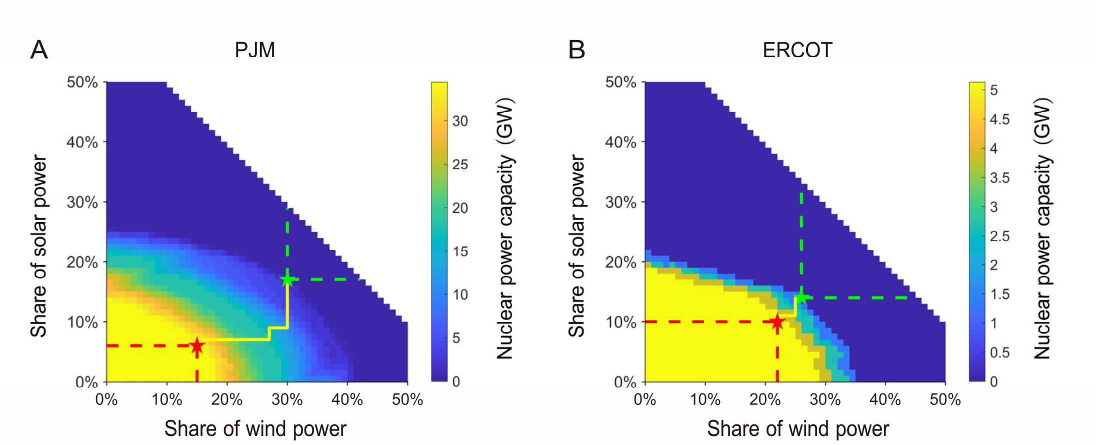


**Figure S2. Nuclear power capacity with different VRE penetration in PJM and ERCOT, related to Figure 3**

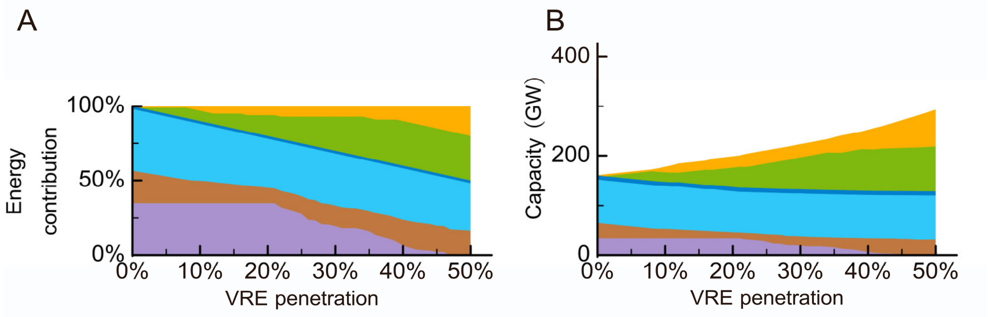


**Figure S3. Change of power generation and capacity on the optimal pathway, related to Figure 3**

As Figure 3, changing the VRE mix before and after VRECON occurs will also affect GHG emissions with the same VRE penetration, which is caused by the change in the proportion of different fossil fuel power generation. The difference, however, is relatively slight than the difference caused by VRECON. For simplicity, we only show the path with the lowest emission.

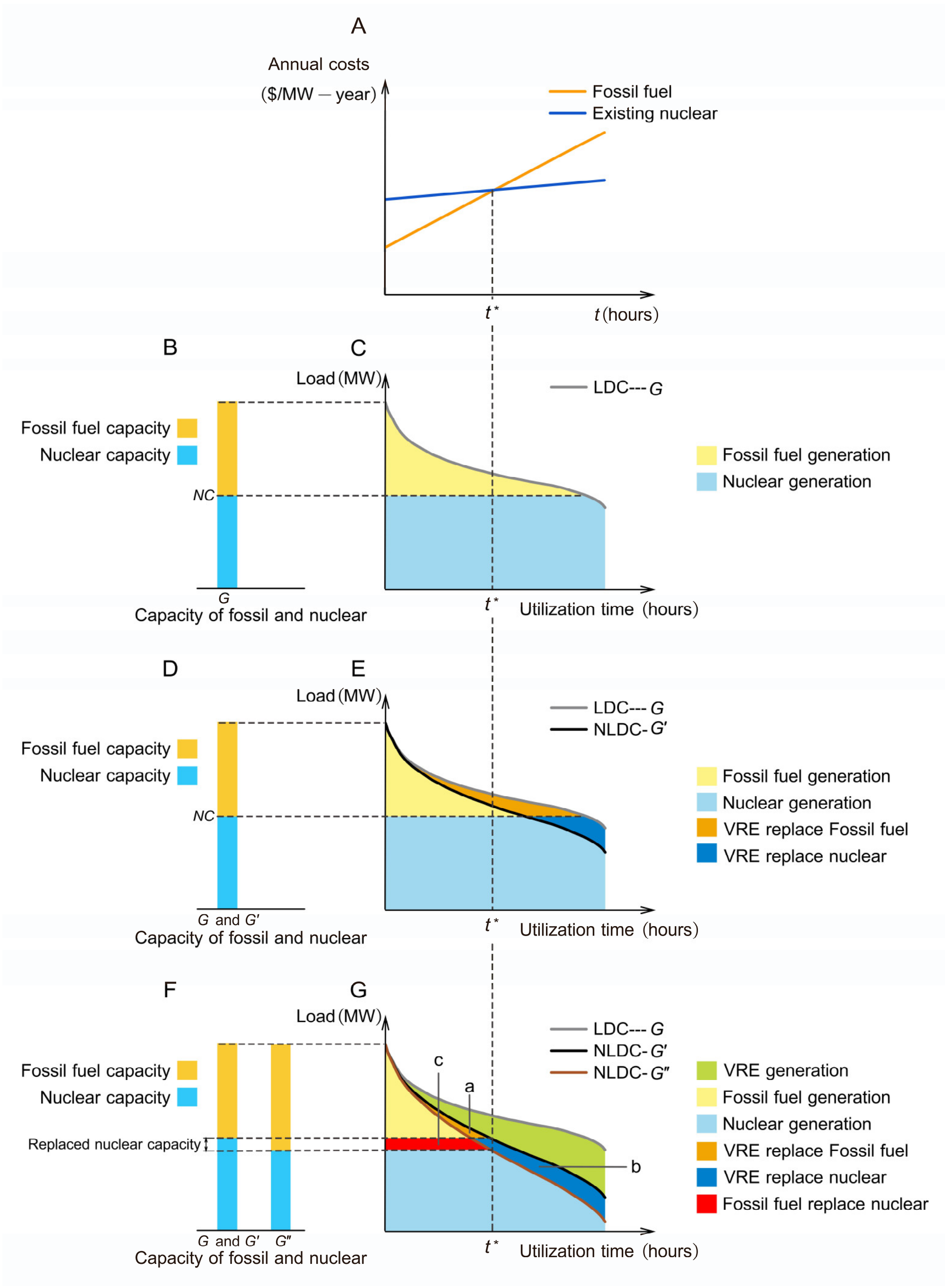


**Figure S4. Analytical framework, related to STAR Methods**

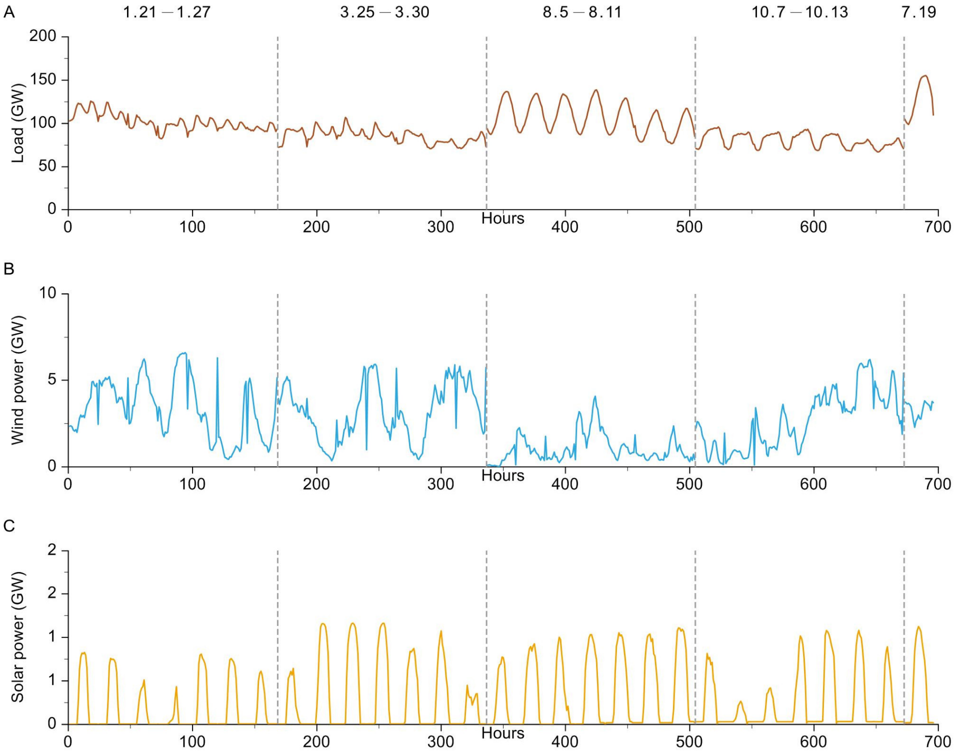


**Figure S5. Selected typical weeks and day in PJM, related to STAR Methods**

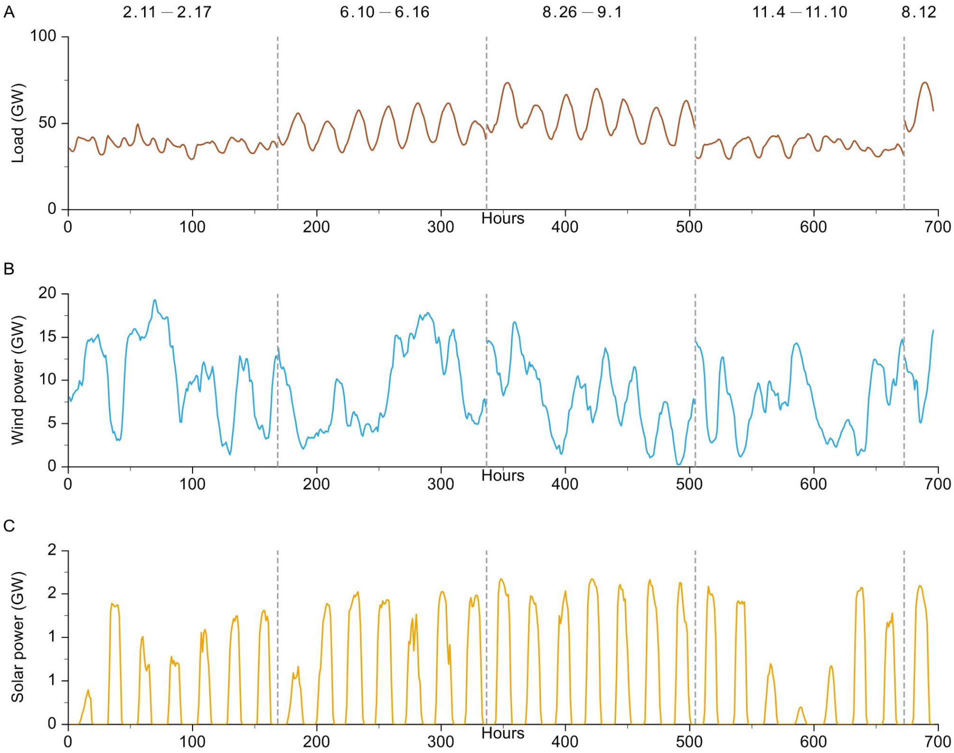


**Figure S6. Selected typical weeks and day in ERCOT, related to STAR Methods**

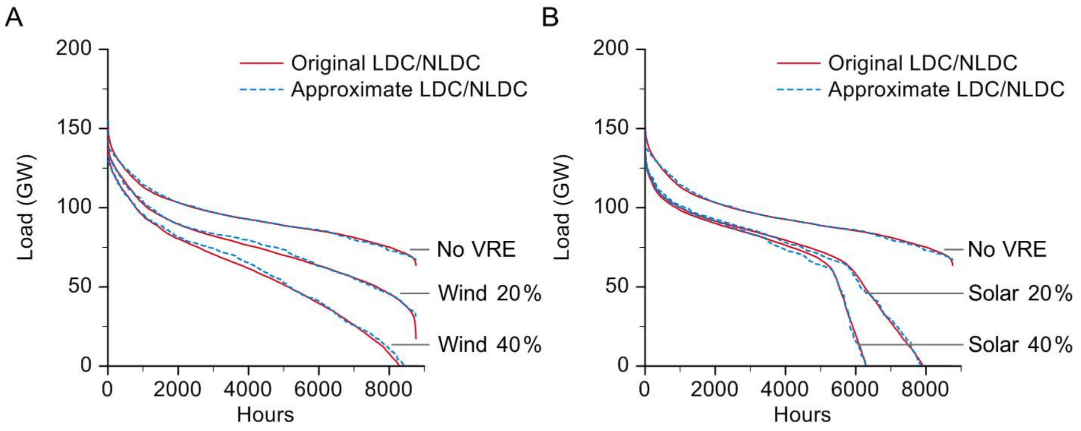


**Figure S7. Graphical representation of the system's LDC, NLDC and their approximations in PJM, related to STAR Methods**

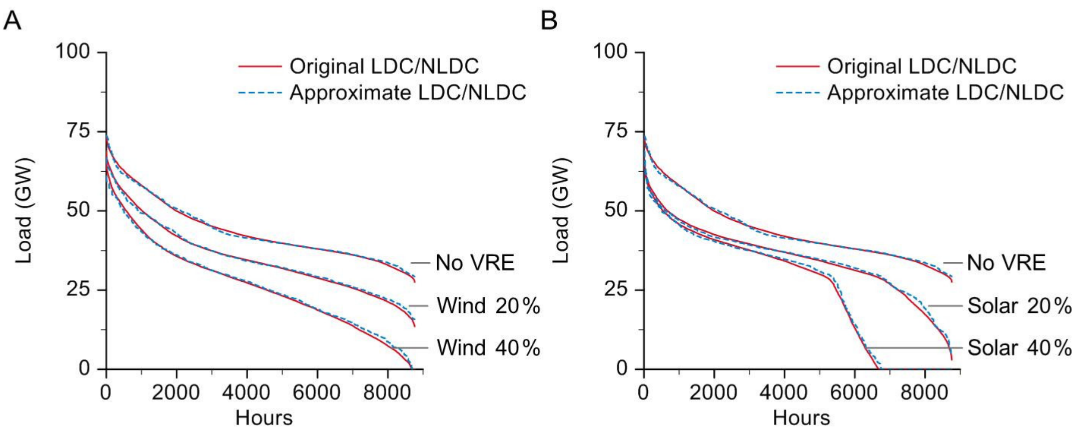


**Figure S8. Graphical representation of the system's LDC, NLDC and their approximations in ERCOT, related to STAR Methods**

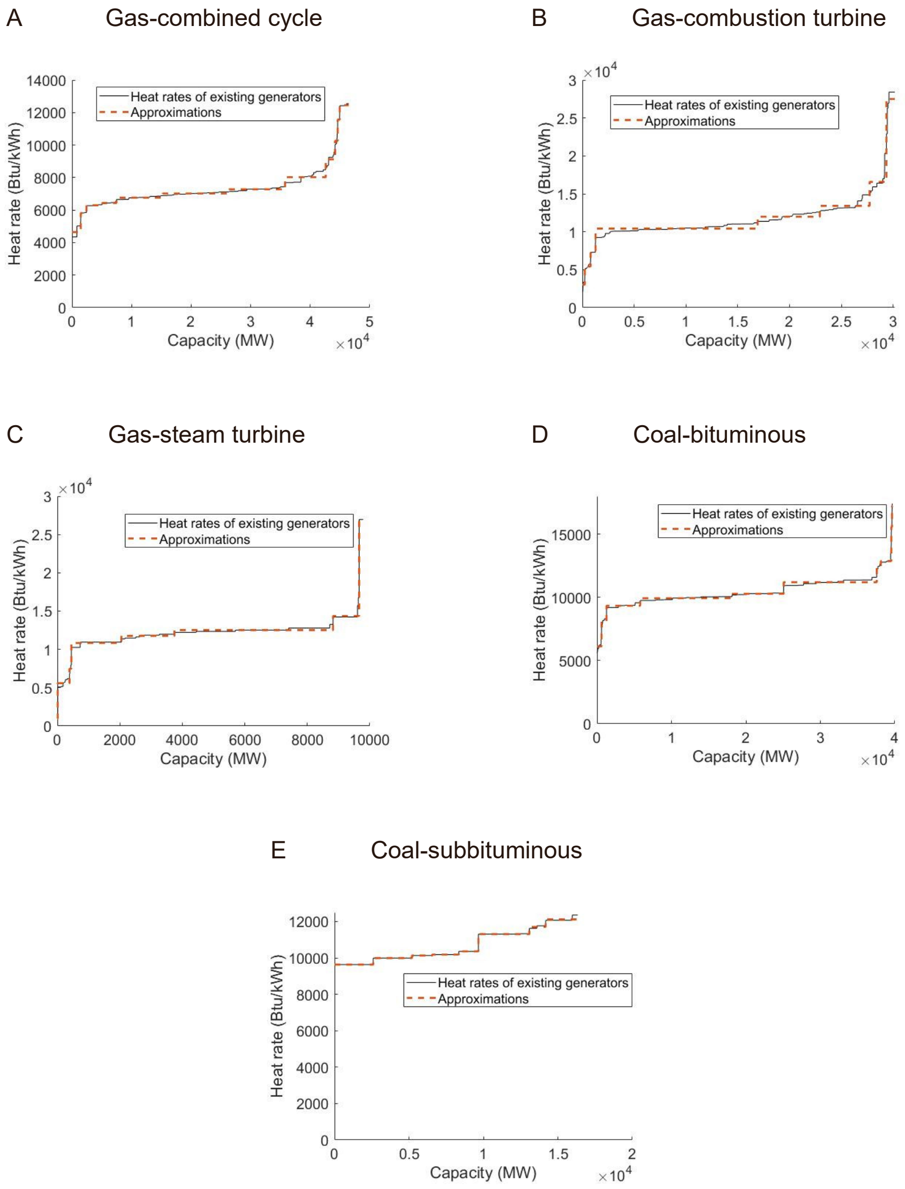


**Figure S9. The heat rates distributions and approximations of existing generators in PJM, related to STAR Methods**

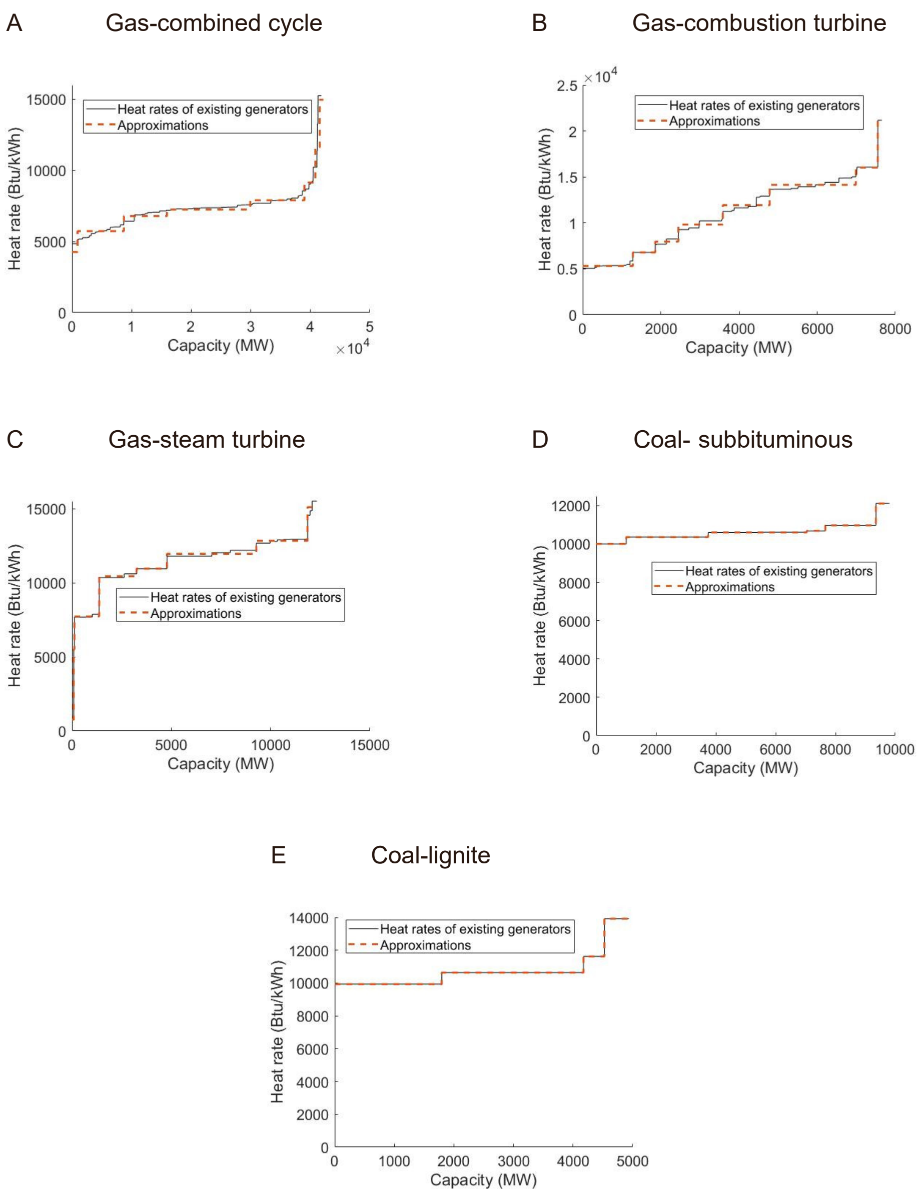


**Figure S10. The heat rates distributions and approximations of existing generators in ERCOT, related to STAR Methods**

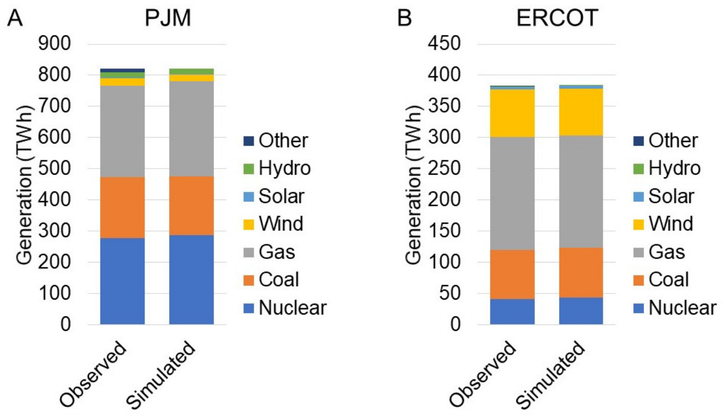


**Figure S11. Actual observation outcomes and simulation results in 2019, related to STAR Methods**

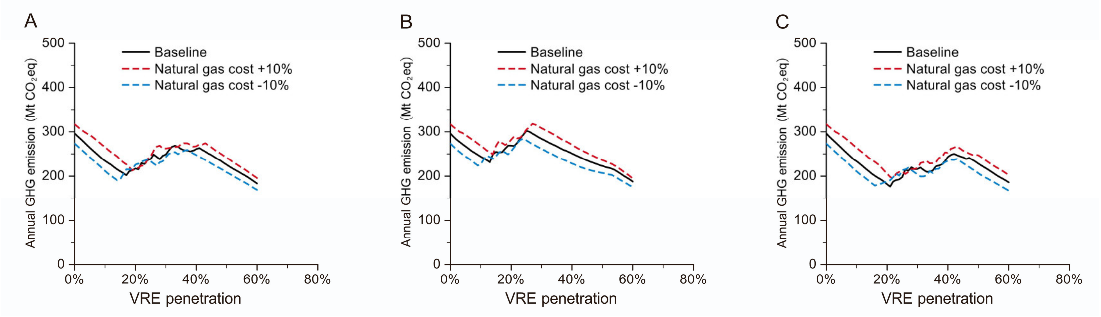


**Figure S12. Annual GHG emissions in the supplemental analysis of natural gas cost, related to STAR Methods**

(A) Wind only.

(B) Solar only.

(C) The optimal pathway.

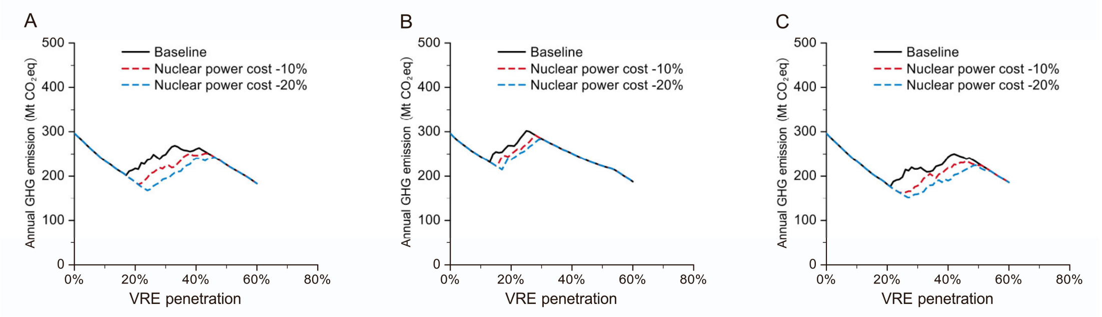


**Figure S13. Annual GHG emissions in the supplemental analysis of nuclear power cost, related to STAR Methods**

(A) Wind only.

(B) Solar only.

(C) The optimal pathway.

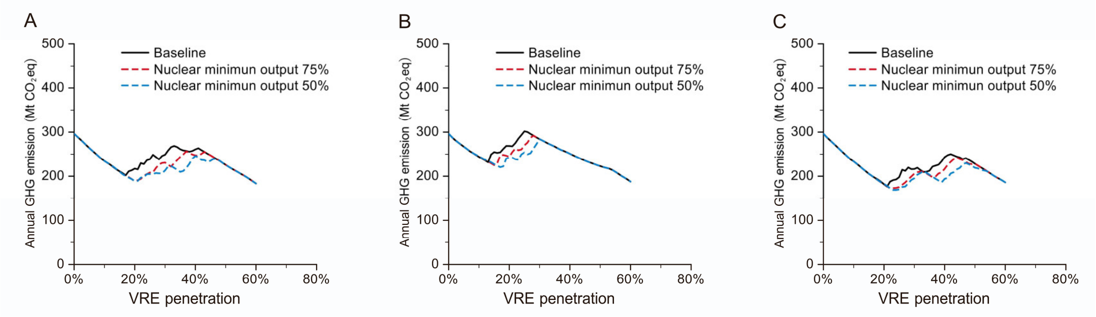


**Figure S14. Annual GHG emissions in the supplemental analysis of nuclear power flexibility, related to STAR Methods**

(A) Wind only.

(B) Solar only.

(C) The optimal pathway.

## Supplemental Tables

### Table S1. Parameters of fossil energy and VRE, related to STAR Methods

| Type | | Fuel costs ($/MMBtu) | Heat rates of new generators (Btu/kWh) | O&M costs | | Start-up costs | | Minimum output (%) | Overnight capital cost ($/kw) | Lifetime (year) |
|---|---|---|---|---|---|---|---|---|---|---|
| | | | | Fixed ($/kW-year) | Variable ($/MWh) | Fuel requirement for cold start ($MWh_{th}$/MW) | Depreciation costs ($/MW) | | | |
| Coal | BIT | 2.25 | 8800 | 42 | 2.66 | 5.9 | 65.2 | 40 | 3636 | 40 |
| | SUB | 1.86 | - | 42 | 2.66 | 5.9 | 65.2 | 40 | - | - |
| | LIG | 1.68 | - | 42 | 3.46 | 5.9 | 65.2 | 40 | - | - |
| Gas | CC | 2.94 | 6600 | 11 | 1.73 | 2.8 | 80.0 | 45 | 978 | 30 |
| | CT | 2.94 | 10000 | 17.5 | 2.00 | 0.1 | 31.9 | 20 | 1101 | 30 |
| | ST | 2.94 | - | 22.1 | 2.00 | 2.8 | 75.8 | 38 | - | - |
| Wind | | - | - | 39.7 | 0 | - | - | 0 | 1575 | 20 |
| Solar | | - | - | 23.4 | 0 | - | - | 0 | 1096 | 20 |

Note: BIT–bituminous, SUB–subbituminous, LIG-lignite, CC-combined cycle, CT-combustion turbine, ST-steam turbine, O&M-Operation and Maintenance.

**Table S2. Parameters of Nuclear power, related to STAR Methods**

| Type | Fuel cost ($/MWh) | Operating cost ($/MWh) |
|---|---|---|
| Pressurized water reactors | 6.20 | 18.00 |
| Boiling water reactors | 6.07 | 19.62 |

**Table S3. GHG emission factors and global warming potential, related to STAR Methods**

| | | $CO_2$ (kg/MMBtu) | $CH_4$ (g/MMBtu) | $N_2O$ (g/MMBtu) |
|---|---|---|---|---|
| Emission factor by fuel type | Bituminous | 93.28 | 11 | 1.6 |
| | Subbituminous | 97.17 | 11 | 1.6 |
| | Lignite | 97.72 | 11 | 1.6 |
| | Natural gas | 53.06 | 1 | 0.1 |
| Global warming potential | | 1 | 25 | 298 |
